# Effect of a health literacy training program for surgical oncologists and specialized nurses on disparities in referral to breast cancer genetic testing

**DOI:** 10.1016/j.breast.2021.04.008

**Published:** 2021-04-22

**Authors:** Jeanine A.M. van der Giessen, Sandra van Dulmen, Mary E. Velthuizen, Maria E.T.C. van den Muijsenbergh, Klaartje van Engelen, Margriet Collée, Thijs van Dalen, Cora M. Aalfs, Maartje J. Hooning, Peter M.M. Spreeuwenberg, Mirjam P. Fransen, Margreet G.E.M. Ausems

**Affiliations:** a Division Laboratories, Pharmacy and Biomedical Genetics, Department of Genetics, University Medical Center Utrecht, Utrecht, the Netherlands; b Nivel (Netherlands Institute for Health Services Research) Utrecht, the Netherlands; c Radboud University Medical Center, Radboud Institute for Health Sciences, Department of Primary and Community Care, Nijmegen, the Netherlands; d Pharos, Center for Healthcare Disparities, Utrecht, the Netherlands; e Department of Clinical Genetics, Amsterdam UMC, VU University Medical Center, Amsterdam, the Netherlands; f Department of Clinical Genetics, Erasmus University, Medical Center, Rotterdam, the Netherlands; g Division of Surgery, Diakonessenhuis Utrecht, the Netherlands; h Department of Clinical Genetics, Amsterdam UMC, Academic Medical Center, Amsterdam, the Netherlands; i Department of Medical Oncology, Erasmus MC Cancer Institute, University Medical Center Rotterdam, the Netherlands; j Department of Public and Occupational Health, Amsterdam Public Health Research Institute, Amsterdam UMC, University of Amsterdam, the Netherlands

**Keywords:** Breast cancer genetic testing, Referral, Access to care, Health literacy, Training program, Communication

## Abstract

**Background:**

There is an underuse of genetic testing in breast cancer patients with a lower level of education, limited health literacy or a migrant background. We aimed to study the effect of a health literacy training program for surgical oncologists and specialized nurses on disparities in referral to genetic testing.

**Methods:**

We conducted a multicenter study in a quasi-experimental pre-post (intervention) design. The intervention consisted of an online module and a group training for surgical oncologists and specialized nurses in three regions in the Netherlands. Six months pre- and 12 months post intervention, clinical geneticists completed a checklist with socio-demographic characteristics including the level of health literacy of each referred patient. We conducted univariate and logistic regression analysis to evaluate the effect of the training program on disparities in referral to genetic testing.

**Results:**

In total, 3179 checklists were completed, of which 1695 were from hospital referrals. No significant differences were found in educational level, level of health literacy and migrant background of patients referred for genetic testing by healthcare professionals working in trained hospitals before (n = 795) and after (n = 409) the intervention. The mean age of patients referred by healthcare professionals from trained hospitals was significantly lower after the intervention (52.0 vs. 49.8, P = 0.003).

**Conclusion:**

The results of our study suggest that the health literacy training program did not decrease disparities in referral to genetic testing. Future research in a more controlled design is needed to better understand how socio-demographic factors influence referral to breast cancer genetic testing and what other factors might contribute.

## Highlights

• Study on the effect of a health literacy training on access to breast cancer genetic testing.
• Background characteristics of breast cancer patients pre- and post-intervention are compared.
• Mean age of patients referred by trained hospitals was significantly lower after intervention.
• The health literacy training did not decrease disparities in referral to genetic testing.

## 1 Introduction

Despite guidelines that recommend genetic testing for patients at increased risk of carrying a pathogenic variant in a breast cancer gene (e.g. *BRCA1*, *BRCA2*, *CHEK2*, *PALB2*, *ATM*) [[1], [2], [3]] not all eligible patients are referred for genetic testing. Previous studies show that patients with a lower level of education or a (non-Western) migrant background have poorer access to genetic testing.

[[4], [5], [6], [7], [8], [9]] These disparities in referral may lead to differences in treatment and survival rates, because early detection of a pathogenic variant has the potential to improve health outcomes [10]. Besides, carrying a *BRCA* pathogenic variant implies a change in follow-up measures as these patients may have an increased risk of developing a second breast cancer or ovarian cancer. The detection of a pathogenic variant enables predictive DNA testing in healthy family members [[11], [12], [13]]. Currently, eligible newly diagnosed breast cancer patients are usually offered rapid genetic testing before their primary surgery [14,15]. These patients mostly have a higher overall genetic testing uptake compared to patients in routine care [16].

Several barriers to genetic testing have been identified, including worries regarding insurance coverage for genetic testing and concerns about misuse of testing, privacy and confidentiality issues [17,18]. In addition, patients with a lower level of education or a migrant background have limited access to genetic testing due to a lack of physician recommendation [5,7,9,19]. Ineffective communication is widely recognized as a major contributor to such health disparities [20]. Patients’ level of health literacy, i.e. the degree to which someone has the capacity to obtain, process, and understand basic health information and services needed to make appropriate health decisions, seems to play an important role [[21], [22], [23], [24], [25], [26]]. Individuals with limited health literacy may understand less of the written and oral communication they receive about genetic information and may participate less in consultations with healthcare professionals [25,27]. They also have less medical knowledge, which might hamper patient-initiated inquiry [28]. Non-cognitive aspects of health literacy, such as motivation and self-confidence, also defined as ‘the capacity to act’, are also likely to have an impact on communication, making it difficult for patients to participate actively in healthcare decisions [29,30]. Among patients with a lower level of education or a migrant background, the level of health literacy is relatively low [31]. Besides, limited language proficiency in turn affects the level of health literacy, reduces access to healthcare systems and leads to poorer health outcomes [32,33].

Surgical oncologists and specialized nurses, the main referrers to genetic testing for patients with breast cancer, may be insufficiently aware of the negative impact of limited health literacy on medical communication [5,34]. They do not recognize limited health literacy in patients and lack the skills needed to effectively discuss (referral to) breast cancer genetic testing [24,35]. We therefore developed a health literacy training program (Erfo4all) for healthcare professionals (i.e. surgical oncologists and specialized nurses involved in breast cancer care), consisting of an online module and a group training on location [36]. In a previous study, the effect of a health literacy program on healthcare professionals’ awareness, knowledge and self-efficacy related to communication about genetic testing with patients with limited health literacy or a migrant background was examined [37]. The program appeared to improve healthcare professionals’ ability to communicate effectively about breast cancer genetic testing with ‘communication-vulnerable’ patients [38,39]. The overall aim of the current study was to evaluate the effect of the health literacy training program on disparities in referral to breast cancer genetic testing. Specific research questions were:

1) What are the background characteristics of all patients referred by healthcare professionals in trained hospitals compared to those of patients referred in untrained hospitals?
2) a) Does the number of patients with a lower level of education, limited health literacy or a migrant background referred by healthcare professionals from trained hospitals differ before and after the health literacy training program?
  b) Do these numbers vary between the rapid genetic testing setting and routine care?

## 2 Methods

### 2.1 Study design

We used a quasi-experimental pre-post (intervention) design to study the effect of the health literacy training program. Healthcare professionals from 19 hospitals (4 academic and 15 non-academic hospitals), who refer patients for breast cancer genetic testing to one of the four university medical centers in three regions in the Netherlands, were invited to participate in a health literacy training program [36,37]. The training program consisted of an online module (18 min) and a group training on location (2h). The online module focused on knowledge acquisition, while in the group training practicing skills were most important [36].

*Participants*

To measure the effect of the health literacy training program on the rates of referral, clinical geneticists and genetic counselors from the four university medical centers were asked to fill in a checklist for all new patients referred for breast cancer genetic testing. They started with the checklist registration approximately 6 months before the training of healthcare professionals (baseline) in their region and continued until 12 months after the training. The total registration period in the study was from March 2017 until March 2019 Inclusion in the pre- or post-intervention group was based on (estimated) date of referral. All breast cancer patients who were treated in academic and non-academic hospitals, and referred for diagnostic genetic testing by their surgical oncologist or specialized nurse, were eligible for the study. Patients referred by their general practitioner were excluded because general practitioners were not invited to the training program. These patients were mainly referred for predictive genetic testing (e.g. testing when a pathogenic variant was detected in an affected family member).

### 2.2 Data collection

*Checklist*

The checklist used in this study was based on previous studies on determinants of referral to breast cancer genetic testing [5,6]. The checklist contained patients’ demographics (i.e. level of education, migrant status, level of health literacy, language proficiency, disease status and referral pathway (i.e. referred by general practitioner or a hospital), referral for diagnostic or predictive DNA testing, and referral for rapid genetic testing or routine care).

Patients’ level of education was determined by the Dutch Standard Classification of Education [40] and the international classification of the UNESCO [41], i.e. lower level of education: (pre-) primary education or first stage of basic education; intermediate-1 educational level: lower secondary or second stage of basic education; intermediate-2 educational level: (upper) secondary education; and higher level of education: tertiary education. The migrant status of the counselee was determined according to the definition of Statistics Netherlands [42]. According to this definition, a patient is a migrant when at least one of their parents was born outside of the Netherlands. Furthermore, a distinction was made between Western migrants (at least one parent born outside the Netherlands, but in Europe, North America, Australia, New Zealand, Indonesia or Japan) and non-Western migrants (at least one parent was born in Turkey or countries in Africa, Latin America or Asia). Because of practical considerations (time constraints) and ethical considerations, it was not possible to ask patients to complete one of the health literacy assessment instruments during the visit at the outpatient clinic, like the Short Test of Functional Health Literacy in Adults (S-TOFHLA) or the Rapid Estimate of Adult Literacy in Medicine (REALM). To still get an indication of the level of health literacy, we choose a valid measurement that was most likely to be applicable in everyday clinical practice. The level of health literacy was assessed by one of the validated screening questions from Chew known to be effective in identifying patients with inadequate health literacy, i.e.: ‘How often do you have someone help you read hospital materials?’ [43].

*Trained hospitals*

**Fig. 1:**
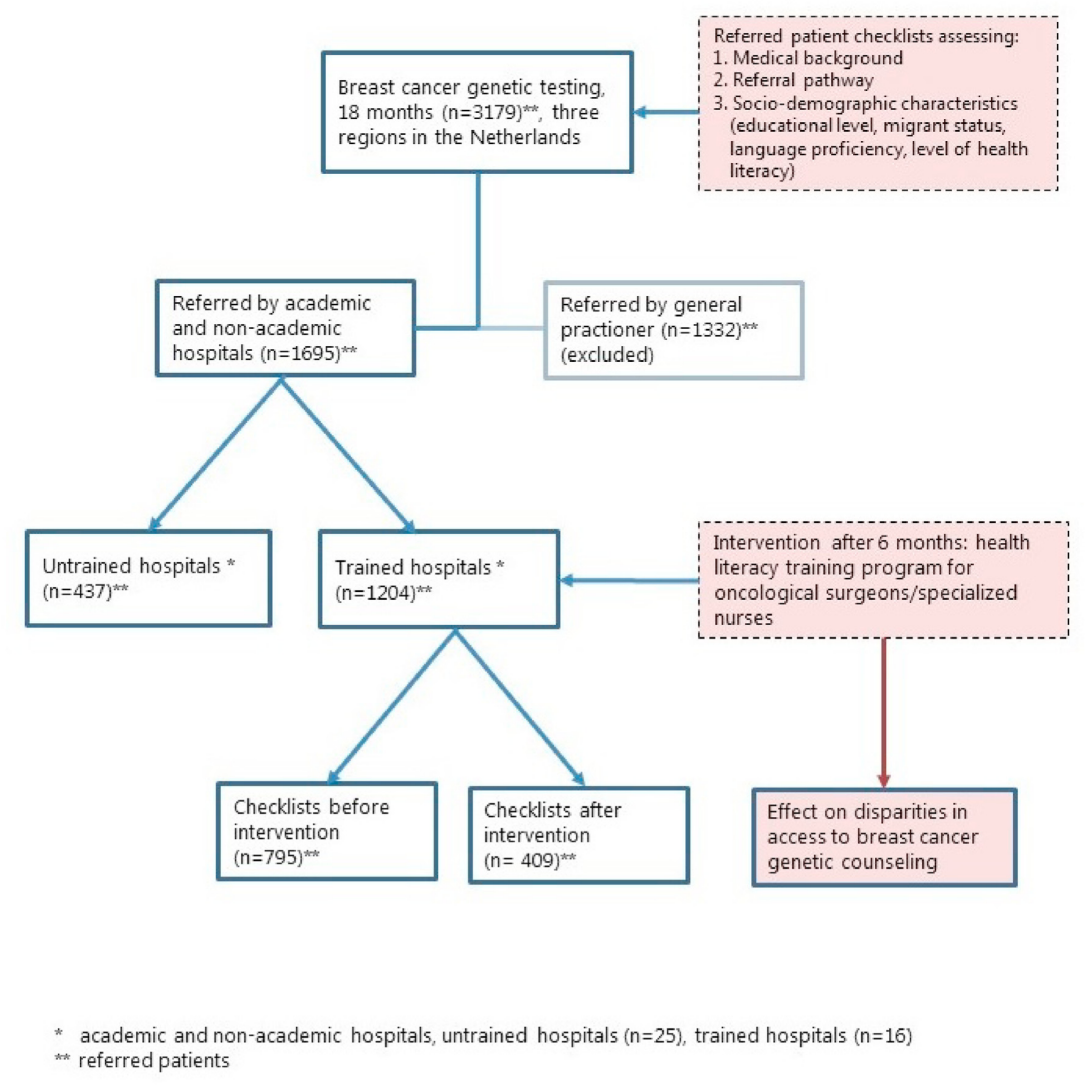
Study design health literacy training program.

A total of 73 healthcare professionals from 19 hospitals that were invited, responded to the invitation. Healthcare professionals (n = 59) from 16 hospitals completed the whole training program. However, not all healthcare professionals working in one of the 16 hospitals and referring patients to breast cancer genetic testing, participated in the training program. We assumed that the trained healthcare professionals shared their learning experience during multidisciplinary meetings and therefore use the term ‘trained hospitals’ to indicate healthcare professionals from hospitals that participated in the training program. We previously showed that more than 41% of the healthcare professionals actually reported to share their experience with their colleagues [37]. Healthcare professionals referring from ‘control’ hospitals are defined as ‘untrained hospitals’ (n = 25). Only patients referred by healthcare professionals in a trained hospital were considered in the analyses for the pre- and post-intervention comparison. In the analysis, we furthermore distinguished between rapid referrals, i.e., early after diagnosis when results are needed for treatment plans, and routine referrals. Due to privacy issues, it was not always possible to know the actual referral date for patients in routine care. When the actual referral date was unknown, the average waiting time during the registration period was imputed to estimate the referral date. Fig. 1 shows the study design.

### 2.3 Statistical analysis

The primary outcome was the percentage of patients referred for breast cancer genetic testing with a lower level of education, limited health literacy or a migrant background. Categorical variables were described as totals and percentages. Continuous variables were described as a mean and standard deviation (SD). Univariate analysis was performed to compare the distribution of patient characteristics before and after the intervention in the trained hospitals, using the independent sample *t*-test for continuous variables, and Chi-square or Fisher’s exact tests for the categorical variables. Patient characteristics included age, breast cancer patient status, migrant status, level of education, and language proficiency. Furthermore, to adjust for potential confounders, such as age, migrant status, referral for rapid counseling and educational level, we performed a multivariate logistic regression. We tested whether it was more likely for women with limited health literacy to be part of the post-intervention group as compared to the pre-intervention group (outcome measure). As language proficiency and limited health literacy were strongly correlated, language proficiency was excluded from the logistic regression model to avoid multi-collinearity. Limited health literacy, with as variable ‘need help reading hospital materials’, was coded as never/once in a while (0) and often/always [1]. All tests were two-sided and p < 0.05 was considered statistically significant. The analyses were conducted with SPSS version 24.0 (IBM Corporation, Armonk, NY, USA).

## 3 Results

### 3.1 Background characteristics of patients referred for genetic testing

Between March 2017 and March 2019, clinical geneticists and genetic counselors completed 3179 checklists. About half of the referred patients (52%) were affected with breast cancer and 53% of all patients were offered predictive DNA testing. Most referrals (56%) came from hospitals, 44% of the patients were referred by their general practitioner and 45% of the hospital referrals concerned rapid genetic testing. Background characteristics of all patients referred by hospitals (n = 1695) showed that the majority of patients had a Dutch background (79%), while 10% of patients had a non-Western migrant background. In total 37% of the patients seen for genetic testing had a high level of education, while 4% had a low level of education. Almost 4% of patients referred by hospitals had low or limited health literacy, and the level of language proficiency was low for 3% of the patients. There were 1204 patients (71%) referred by healthcare professionals from trained hospitals, and 437 patients (26%) by untrained hospitals. We found no differences in background characteristics of patients between the three regions (Utrecht, Amsterdam, Rotterdam). Table 1 shows the background characteristics of all patients referred for genetic testing and those referred by trained and untrained hospitals.

**Table 1:** Characteristics counselees requesting breast cancer genetic counseling.

	All counselees		Hospital referrals^a^						
			Total			trained hospitals		untrained hospitals	
	N	%	N	%		N	%	N	%
total^b^	3179		1695						
total excluding missing trained status	–		1641			1204	71.0%	437	25.8%
mean age referral	48.9		51.6			51.2		52.8	
(min-max)	(18–92)		(18–88)			(18–88)		(23–83)	
gender	3179		1695			1204		437	
male	294	9.2%	36		2.1%	29	2.4%	7	1.6%
female	2885	90.8%	1659		97.9%	1175	97.6%	430	98.4%
breast cancer	3179		1641			1204		437	
affected	1641	51.6%	1417		86.3%	1024	85.0%	393	89.9%
unaffected	1538	48.4%	224		13.7%	180	15.0%	44	10.1%
mean age breast cancer	49.3		49.2			49.1		49.6	
(min-max)	(24–87)		(24–87)			(24–87)		(27–83)	
DNA-testing	3010		1535			1132		403	
diagnostic	1421	47.2%	1178		76.6%	833	73.6%	345	85.6%
predictive	1589	52.8%	357		23.3%	299	26.4%	58	14.4%
rapid DNA-testing	3140		1614			1191		423	
yes	662	21.1%	627		44.6%	475	39.9%	152	35.9%
no	2478	78.9%	987		55.4%	716	60.1%	271	64.1%
educational level	3021		1668			1148		417	
low	103	3.4%	61		3.9%	45	3.9%	16	3.8%
intermediate-I	616	20.4%	353		22.6%	233	20.3%	120	28.8%
intermediate-II	1097	36.3%	566		36.2%	404	35.2%	162	38.8%
high	1205	39.9%	585		37.4%	466	40.6%	119	28.5%
need help because of limited HL	3133		1614			1184		430	
never	2825	90.2%	1448		89.7%	1052	88.9%	396	92.1%
once in a while	199	6.4%	105		6.5%	87	7.3%	18	4.2%
often	47	1.5%	26		1.6%	19	1.6%	7	1.6%
always	62	2.0%	35		2.2%	26	2.2%	9	2.1%
language proficiency	3148		1624			1193		431	
good/intermediate proficiency	3057	97.1%	1574		96.9%	1151	96.5%	423	98.0%
low proficiency	52	1.6%	28		1.7%	24	2.0%	4	1.0%
no proficiency	39	1.2%	22		1.4%	18	1.5%	4	1.0%
migrant status counselee	3146		1623			1192		431	
Dutch native	2550	81.1%	1289		79.4%	927	77.7%	362	84.0%
migrant	596	18.9%	334		20.6%	265	22.2%	69	16.0%
country of origin known	575		324			259		65	
• western	307	9.8%	164		10.0%	134	11.2%	30	6.7%
• non western	268	8.5%	160		9.6%	125	10.5%	35	8.1%
interpretor present	3150		1625			1193		432	
No	3100	98.4%	1593		98.0%	1169	98.0%	424	98.1%
Yes	50	1.6%	32		2.0%	24	2.0%	8	1.9%
• family	42	87.5%	29		90.6%	21	87.5%	0	0.0%
• professional	6	12.4%	3		9.4%	3	12.5%	8	100.0%

a Excluding records training unknown.

b Hospital referrals n = 1695 (53%), general practitioner referrals n = 1332 (42%), unknown n = 152 (5%).

### 3.2 Effect of the health literacy training program on disparities in referral to breast cancer genetic testing in routine care and rapid genetic testing

For 729 patients in the Utrecht region the date of referral could be retrieved. For 966 patients from the other two regions, we could only register the week or month of first consultation at the genetics department. Among the 1204 breast cancer patients referred by healthcare professionals in trained hospitals, 795 (66%) breast cancer patients were referred before the intervention and 409 (44%) after the intervention. In the univariate analysis for the pre- and post-intervention groups, no significant association was found between migrant status, level of education, or level of health literacy and the intervention.

Looking at health literacy, we found that 89 (11.4%) breast cancer patients with low or limited health literacy are referred before the intervention and 43 (10.7%) were referred after the intervention. Moreover, multivariate regression analysis showed no effect on referral to genetic testing of patients with limited health literacy after the introduction of the health literacy training program (OR = 0.399, 95% CI = 0.156–1.021), after adjusting for potential confounding factors such as age, migrant status, referral for rapid counseling and level of education. Moreover, no difference was found in the separate analyses between rapid genetic testing only (OR = 0.69, 95% CI = 0.25–1.92) and routine care only (OR = 0.69, 95% CI = 0.27–1.74). In addition, lower age was statistically significantly associated with the intervention, indicating that younger patients were more likely to be referred for genetic testing after the intervention (p = 0.003). This effect was not found in patients who underwent rapid genetic counseling. Table 2 shows pre- and post-intervention results for all patients referred by healthcare professionals from trained hospitals and the results of patients referred for rapid genetic testing and in routine care.

**Table 2:** Pre- and post intervention results patients referred by trained hospitals (n = 1204).

Variable		Total		Before intervention		After intervention		p
		N	%	N	%	N	%	
**ALL REFERRALS TRAINED HOSPITALS**								
Total		1204		795		409		
mean age referral (min – max)		51.2 (18–88)		52.0 (18–88)		49.8 (20–87)		**0.003∗**
affected (N = 1204)	yes	1024	85%	680	85.5%	344	84.1%	**n.s.**
	no	180	15%	115	14.5%	65	15.9%	
migrant status counselee (N = 1192)	Dutch native	927	77.8%	610	77.8%	317	77.5%	**n.s.**
	migrant • western • non western • unknown	265	22.2%	173	22.2%	92	22.5%	**n.s.**
		134	50.6%	80	46.2%	54	58.7%	
		125	47.2%	89	51.4%	36	39.1%	
		6	2.3%	4	2.3%	2	2.2%	
educational level (N = 1148)	low	45	3.9%	33	4.4%	12	3.0%	**n.s.**
	intermediate-I	233	20.3%	163	21.6%	70	17.7%	
	intermediate-II	404	35.2%	264	35.1%	140	35.4%	
	high	466	40.6%	293	38.9%	173	43.8%	
need help because of limited HL (N = 1184)	never	1052	88.9%	694	88.6%	358	89.3%	**n.s.**
	once in a while	87	7.3%	57	7.3%	30	7.5%	
	often	19	1.6%	11	1.4%	8	2.0%	
	always	26	2.2%	21	2.7%	5	1.2%	
language proficiency (N = 1193)	good/fair	1151	96.5%	756	96.2%	395	97.1%	**n.s.**
	bad	24	2.0%	17	2.2%	7	1.7%	
	none	18	1.5%	13	1.7%	5	1.2%	
interpretor present	no	1169	98,0%	768	98.0%	401	98.0%	**n.s.**
(N = 1193)	yes	24	2.0%	16	2.0%	8	2.0%	
(family or professional known N = 24)	• family	21	87.5%	14	87.5%	7	87.5%	
	• professional	3	12.5%	2	12.5%	1	12.5%	
**RAPID GENETIC COUNSELING ONLY**								
Total		475		302		173		
mean age referral (min – max)		46.3 (18–76)		46.3 (18–75)		46.4 (25–76)		**n.s.**
migrant status counselee (N = 469)	Dutch native	355	75.7%	226	76.4%	129	76.4%	**n.s.**
	migrant • western • non western	114	24.3%	70	23.6%	44	25.4%	
		40	8.5%	18	3.8%	22	4.7%	
		70	14.9%	49	10.4%	21	4.4%	
educational level (N = 454)	low	15	3.3%	11	3.9%	4	2.4%	**n.s**.
	intermediate-I	81	17.8%	50	17.5%	31	18.3%	
	intermediate-II	153	33.7%	91	31.9%	62	36.7%	
	high	205	45.2%	133	46.7%	72	42.6%	
need help because of limited HL (N = 465)	never	408	87.8%	261	88.5%	147	865%	**n.s.**
	once in a while	38	8.2%	21	7.1%	17	10.0%	
	often	7	1.5%	4	1.4%	3	1.8%	
	always	12	2.6%	9	3.1%	3	1.8%	
language proficiency (N = 469)	good/fair	455	97.0%	287	96.6%	168	97.7%	**n.s.**
	bad	6	1.3%	4	1.3%	2	1.2%	
	none	8	1.7%	6	2.0%	2	1.2%	
interpretor present	no	461	98.1%	291	98.0%	170	98.3%	**n.s.**
(N = 470)	yes	9	1.9%	6	2.0%	3	1.7%	
(family or professional known N = 9)	• family	8	88.9%	5	83.3%	3	100.0%	
	• professional	1	11.1%	1	16.7%	0	0.0%	
**NON-RAPID REFERRALS ONLY**								
Total		716		486		230		
mean age referral (min – max)		54.6 (20–88)		55.6 (22–88)		52.5 (20–87)		**0.002∗**
migrant status counselee (N = 710)	Dutch native	561	79.0%	378	78.8%	183	79.6%	**n.s.**
	migrant • western • non western	149	21.0%	102	21.3%	47	20.4%	
		94	13.2%	62	8.7%	32	4.5%	
		53	7.3%	39	5.5%	14	2.0%	
educational level (N = 682)	low	30	4.4%	22	4.8%	8	3.6%	**n.s.**
	intermediate-I	149	21.8%	110	23.8%	39	17.7%	
	intermediate-II	245	35.9%	170	36.8%	75	34.1%	
	high	258	37.8%	160	34.6%	98	34.6%	
need help because of limited HL (N = 706)	never	632	89.5%	427	88.8%	205	91.1%	**n.s.**
	once in a while	48	6.8%	35	7.3%	13	5.8%	
	often	12	1.7%	7	1.5%	5	2.2%	
	always	14	2.0%	12	2.5%	2	0.9%	
language proficiency (N = 711)	good/fair	683	96.1%	462	95.9%	221	96.5%	**n.s.**
	bad	18	2.5%	13	2.7%	5	2.2.%	
	none	10	1.4%	7	1.5%	3	1.3%	
interpretor present	no	695	97.9%	470	97.9%	225	97.8%	**n.s**
(N = 710)	yes	15	2.1%	10	2.1%	5	2.2%	
(family or professional known (N = 15)	• family	13	86.7%	9	90.0%	4	80.0%	
	• professional	2	13.3%	1	10.0%	1	20.0%	

### 3.3 Unexpected results

Due to the significant increase in the self-efficacy of the trained healthcare professionals to communicate effectively with patients with limited health literacy or a migrant background found previously [37], we were surprised that our current study showed no effect on the referral rate of these groups of patients. As sample bias might have been introduced in the pre-intervention group, we conducted an additional logistic regression analysis with untrained hospitals as a second pre-intervention group. With data from this additional analysis, the referral rate of migrant patients tended to be higher (p = 0.063) in trained hospitals after the intervention as compared to referral rate in untrained hospitals. Table 3 shows the result of the logistic regression analyses with patients referred by trained hospitals as the pre-intervention group and the additional logistic regression analysis with patients referred by untrained hospitals as the pre-intervention group.

**Table 3:** Logistic regression trained hospitals and untrained hospitals as pre-intervention group.

	Pre-and post- intervention trained hospitals			Untrained hospitals as pre-intervention group		
	odds ratio	95% CI	p-value	odds ratio	95% CI	p-value
**Variable**						
health literacy	0.399	0.156–1.021	0.055	0.707	0.217–2.307	0.565
migrant status	1.113	0.816–1.517	0.500	1.428	0.981–2.080	0.063
rapid genetic counseling	0.906	0.696–1.179	0.461	0.883	0.653–1.194	0.419
mean age referral	0.988	0.977–0.999	0.026∗	0.984	0.984–0.996	0.010∗

## 4 Conclusions

Our study did not find an effect of a health literacy training program for surgical oncologists and specialized nurses on disparities in referral of patients with a lower level of education, limited health literacy or a migrant background. There were no differences in referral in the rapid genetic counseling setting. In general, the uptake in this setting is already higher compared to routine care because a DNA test may influence surgical treatment decisions. Healthcare professionals believe that rapid genetic testing is beneficial for patients and therefore the tendency to refer eligible patients might be higher [11].

An important finding of our study was that the health literacy training program could make a difference for younger patients with breast cancer in routine care. Referral for the group of younger patients is important because young age at diagnosis of breast cancer indicates a higher risk to carry a *BRCA1* or *BRCA2* pathogenic variant and is a clear indication for referral to breast cancer genetic testing [44]. Despite this, physicians do not systematically discuss genetic testing with young women with breast cancer [8,45]. Therefore, there was extra attention in the training program for the importance of the referral of young (migrant) patients with breast cancer.

Our study has some clear strengths. We conducted a multicenter study, and the involvement of different genetic departments in three regions in the Netherlands increased the generalizability of our study. Further, we included almost 3200 checklists with medical and socio-demographic information of breast cancer patients, of which 1695 checklists (from hospital referrals) are included in analysis. This large sample size is large enough to draw conclusions.

Next to the strengths, there are limitations. It is important to reconsider the study design of the health literacy training program. The most important limitation of our study is the fact that it is unknown which patients are **not** referred during the registration period. We could only register the percentage of referred patients with a lower level of education, limited health literacy or a migrant background. This makes a difference in interpreting the results. Second, based on practical and ethical implications, it was not possible to register for each counselee the healthcare professional who referred the patient to the department of genetics. Instead we used the hospital (trained or untrained) as an independent variable. Third, due to the relatively small number of patients with limited health literacy and a migrant background, there might be a sample fluctuation of patients referred by trained hospitals that are included in the study. The additional logistic regression analysis confirmed that the pre-intervention group might not be representative, which may (partly) explain the unexpected results of our study. Next, the exact date of referral was unknown for patients referred in two regions, so we could not conclude with 100% certainty that referral took place before the intervention. To correct for this omission, we imputed the referral date based on average waiting time. Finally, we used the validated question of Chew (i.e., ‘How often do you have someone help you read hospital materials?) as a self-reported measure to determine the level of health literacy. Although Chew showed that this single question may identify individuals with inadequate health literacy [43], respondents may have given socially desirable answers or may have been too embarrassed to admit that help is needed with reading or interpreting medical information.

### 4.1 Implications for future research

Future research, using a more controlled design, with a larger sample size of patients with limited health literacy or a migrant background is needed to further investigate disparities in referral to breast cancer genetic testing. Furthermore, valid measurement of patient’s level of health literacy is important. For healthcare professionals, being able to correctly assess the patient’s level of health literacy is a prerequisite for effective communication.

Next to factors on the side of the healthcare professionals, like competences to communicate effectively with patients with limited health literacy, other factors might contribute to referral to genetic counseling. Patient’s request, for example, also impacts the referral to breast cancer genetic counseling. Yet, taking the initiative for referral is difficult for patients with limited health literacy. They more often consent to providers’ recommendation [46].

Despite the fact that our study showed no effect on referral to breast cancer genetic testing, we believe in the importance of effective communication and improving the communication skills of healthcare professionals. For all interventions designed to reduce disparities in access to genetic testing and testing, communication about genetic testing in a comprehensible way, for instance by using plain language and using the teach-back method, is an important condition [47,48]. Especially when genetic testing becomes part of mainstream medicine – with the potential to make genetic services accessible to all eligible patients – adapting communication about genetic testing to patients’ needs and abilities is even more essential [49].

## Funding

This study is funded by the Pink Ribbon Foundation, with a donation from Vriendenloterij. Pink Ribbon project number: 2016–204.

## Compliance with ethical standards

No ethical approval was required. The Medical Ethical Committee of the University Medical Center Utrecht considered the Dutch Medical Research involving Human Subjects Act, not applicable to this study.

## Declaration of competing interest

The authors declare no conflict of interest.
